# TBDBT: A TB DataBase Template for collection of harmonized TB clinical research data in REDCap, facilitating data standardisation for inter-study comparison and meta-analyses

**DOI:** 10.1371/journal.pone.0249165

**Published:** 2021-03-26

**Authors:** Taryn Allie, Amanda Jackson, Jon Ambler, Katherine Johnston, Elsa Du Bruyn, Charlotte Schultz, Linda Boloko, Sean Wasserman, Angharad Davis, Graeme Meintjes, Robert J. Wilkinson, Nicki Tiffin

**Affiliations:** 1 Wellcome Centre for Infectious Diseases Research in Africa, Institute of Infectious Disease and Molecular Medicine, University of Cape Town, Cape Town, South Africa; 2 Computational Biology Division, Integrative Biomedical Sciences, University of Cape Town, Cape Town, South Africa; 3 Department of Medicine, University of Cape Town, Cape Town, South Africa; 4 Department Infectious Diseases, Imperial College London, London, United Kingdom; 5 Francis Crick Institute, London, United Kingdom; 6 Centre for Infectious Disease Epidemiology Research, School of Public Health and Family Medicine, University of Cape Town, Cape Town, South Africa; Jamia Hamdard, INDIA

## Abstract

Clinical tuberculosis research, both within research groups and across research ecosystems, is often undertaken in isolation using bespoke data collection platforms and applying differing data conventions. This failure to harmonise clinical phenotype data or apply standardised data collection and storage standards in turn limits the opportunity to undertake meta-analyses using data generated across multiple research projects for the same research domain. We have developed the Tuberculosis DataBase Template (TBDBT), a template for the well-supported, free and commonly deployed clinical databasing platform, REDCap. This template can be used to set up a new tuberculosis research database with a built-in set of standardised data conventions, to ensure standardised data capture across research projects and programs. A modular design enables researchers to implement only the modules of the database template that are appropriate for their particular study. The template includes core modules for informed consent data, participant demographics, clinical symptoms and presentation, diagnostic imaging and laboratory tests. Optional modules have been designed for visit scheduling and calendar functionality, clinical trial randomisation, study logistics and operations, and pharmacokinetic data. Additional fields can be added as needed. This REDCap template can facilitate collection of high-quality data for tuberculosis research, providing a tool to ensure better data harmonisation, analysis and meta-analysis.

## Introduction

Clinical research in tuberculosis (TB) is a rich and diverse field, and many research projects overlap, collecting and analysing the same types of information in different ways, with research groups or research collaboratives generally working on their own data collection platforms using niche or bespoke methods. There are many rich data sets that could potentially be combined for meta-analysis where appropriate secondary use consent is in place, but such a meta-analysis would prove challenging due to the different data formats, coding and structures. The unique conventions applied within each data collection platform limit what data can be compared between studies—and meta-analyses would therefore require extensive data transformation, cleaning and harmonisation accordingly, prior to analysis.

Whilst curated online data resources may contain harmonised, cleaned and standardised data for specialised types of clinical TB data [1, 2], clinical TB research projects are usually initiated by clinician researchers, with data clerks, managers and analysts to capture and store new data collected on case report forms (CRFs) in the field or laboratory. The aim of this project is to provide a generic TB databasing tool that will assist researchers to build a TB clinical research database for a variety of types of data, with inbuilt data standards and data compatibility with other study data that have been captured in databases built using the same template. In other words, a universal, standardised or unified way of describing, capturing or storing those TB data with a database. To achieve this, we have identified, characterised and standardised essential TB research elements, and developed a standardized harmonized database template as a base on which to develop data collection, storage and analysis.

This database template addresses standardisation at four levels (Table 1): ‘Description’—the way the data element, or ‘field’, is described and interpreted; ‘Field name’—the way the data element is named; ‘Type of Data’—the data type that is captured; and ‘Data Coding’—how it is coded, for example an integer, or a selection from a defined list.

**Table 1:** Examples of levels of standardisation in TBDBT, for patient age, gender, and date of diagnosis.

Description	Field Name	Type of Data	Data Coding
Symptom e.g. cough	Cough	Binary	1, Yes| 0, No
Standardised codes	Sex	Dropdown	1, Male|2, Female|90, Rather not say |99, Other|
Age derived from DoB	Age	Calculated field	rounddown(datediff(’today’,[dob],’y’,’dmy’))
Formatted field	Date of diagnosis	Date	date_dmy

## Methods and results

In recognition of the different types of data that may be collected for different studies, we have used a modular approach so that a researcher wishing to build their own TB clinical research database using this template will be able to select only the modules that are relevant to their specific study. A module can be thought of as a standardized part or independent unit incorporated into a complex structure, namely the database template. An example of data elements grouped together in such a way to form a module would be: observations, symptoms, medical history, clinical assessments, pharmacokinetics would form parts of the medical information module. By developing a REDCap template [3, 4], rather than an actual database, we have provided a tool that can be used for each researcher to set up their own database and extend or modify as needed–there is no centralised database or data storage envisaged. Any data sharing for subsequent meta-analyses would require specific data-sharing agreements to be set up by collaborating parties. The database template presented here instead intends to ensure that at the time of joining datasets under a data-sharing agreement, the datasets will be largely compatible with each other and combining datasets will be simple and accurate.

### Initial assessment and scoping exercise

The initial needs assessment involved investigating existing clinical research data standards and the use of ontologies [5]—standardised codes and descriptions that are machine-readable—linked to TB disease, through review of known standards such as LOINC, CDISC, CDASH, ATC and ICD10 standards. These ontologies are highly specific for data elements in clinical research, but do not provide the user with practical guidance in aligning the ontology codes with database coding standards specifically designed for TB clinical research. There are also TB CRFs published online, but not all are freely available for re-use where the copyright is held by the relevant institution. Some examples of TB research CRFs can be found in the WHO framework for TB [6], TB RePORT international [7], chip (Centre of Excellence for Health, Immunity and Infections) TB:HIV forms [8], Challenge TB tools [9] and NICD notification forms [10].

We reviewed the REDCap curated library of data collection instruments which can be used by researchers at partner institutions, provided citation is declared. However, we found that these instruments do not form a cohesive picture of the TB disease profile as they are limited to bespoke and protocol-specific outcomes, making reuse and generalised repurposing difficult. Examples are described by Obeid *et al*. in [11].

In order to compile a set of specifications for the new, generaliseable TB database template, a panel of CIDRI-Africa TB clinical researchers communicated their requirements for essential clinical research data of the TB disease profile, and described types of data used in research projects in this field. This was used to defined which data are considered essential, and the broad categories that could be interpreted as a module, and informed the next process to identify the scope to each module using a question and answer framework.

In addition to investigating external resources, internal database- and CRF- resources available within the research organisation were reviewed. In order to standardize question-and-answer sets, commonalties across the different studies were mapped. Rules for data conventions, including formats, codes and missing data, were recorded in order to apply these consistently when subsequently building data collection instruments in REDCap (Table 2). A data collection instrument is the REDCap equivalent of a CRF or survey or form that serves as the space where data are input.

**Table 2:** A. Example of coding conventions, and B. List of commonly used codes.

**A. Coding conventions**		
Yes or No		1, Yes| 0, No
Male or Female		1, Male| 2, Female
Negative or Positive		1, Negative | 2, Positive
**B. Common use codes**		
Z89	Unsure	
Z90	Rather not say	
Z91	No result	
Z92	Unable to test	
Z93	Not tested	
Z94	Not available	
Z95	Not done	
Z96	None	
Z97	Not applicable	
Z98	Unknown	
Z99	Other	

Additional necessary characteristics for the database included that it had to be robust, user friendly, modular, highly customizable and quickly scalable. Consideration was also given to i) offline data entry vs online data entry, ii) single data entry vs dual data entry, and iii) the limitations within the version of REDCap in current use at the CIDRI-Africa research centre.

### Implementation in REDCap

Given the above mentioned considerations, we used online, single data entry limited to REDCap version 8.4.3 which were in use at CIDRI AFRICA at the time of building the template. The set of standard questions and answers, mapped codes, data conventions and database considerations were fleshed out, and then used to building the template in REDCap.

Instruments are mapped to events as a way of choosing the appropriate modules. This can be found under the ‘Designate instruments for My Events’ section in REDCap. Events are synonymous with study visits. Repeat events, where similar sets of instruments are grouped together and repeated, can be set up as a generic follow-up visit. In the case on logs where one requires repeated single records with an instrument one could set-up that specific instrument to repeat as many times as needed. This allowed for us to mimic conventional logs of an ongoing nature within REDCap.

This modular approach affords easier management of end-user access roles. For example, clinical observations, processes and procedures that are performed by nurses can be encapsulated; likewise, for doctors, pharmacists, laboratory staff. This allows for focussed user access aligning with data governance best practices. Thus relevant sections on an instrument pertaining to specific roles in the research study could be accessed without requiring scrolling through multitudes of non-relevant fields before reaching the appropriate section.

Skip logic has been applied with leading questions so that whole sections do not have to be completed unless necessary. For example, if an assessment by the doctor rules out cardiac problems one would mark the leading question appropriately and not need to spend time on additional cardiac questions solely for the sake of completeness. Though in a paper-based research projects one would need to add prompts as guidance for researchers filling out the CRFs.

REDCap offers a library of external modules to further enrich research projects. These are custom features and work like add-ons or extensions to a specific project e.g. addition of data visualisations. These were not included in this template as to avoid any compatibility issues, but can subsequently be added to further enhance the functionality of the template in the context of specific research settings.

### Set-up

Guidance documents for the set-up can be found on GitHub at the following link https://github.com/CIDRI-Africa/TBDBT/. The template XML file should be imported into REDCap as a standalone project that does not hold any data. Thereafter duplicates of the template can be deployed as required. Detailed instructions regarding the deployment of the TBDBT in an existing REDCap environment, and which settings need some further fine-tuning thereafter, are provided in the supplementary data viz. ‘Set-up guide’.

### Maintenance

Versioning is not automatically updated within the project. The database administrator or manager of each project is required to make the necessary changes as the research project progresses.

### Capturing informed consent data

**Fig 1:**
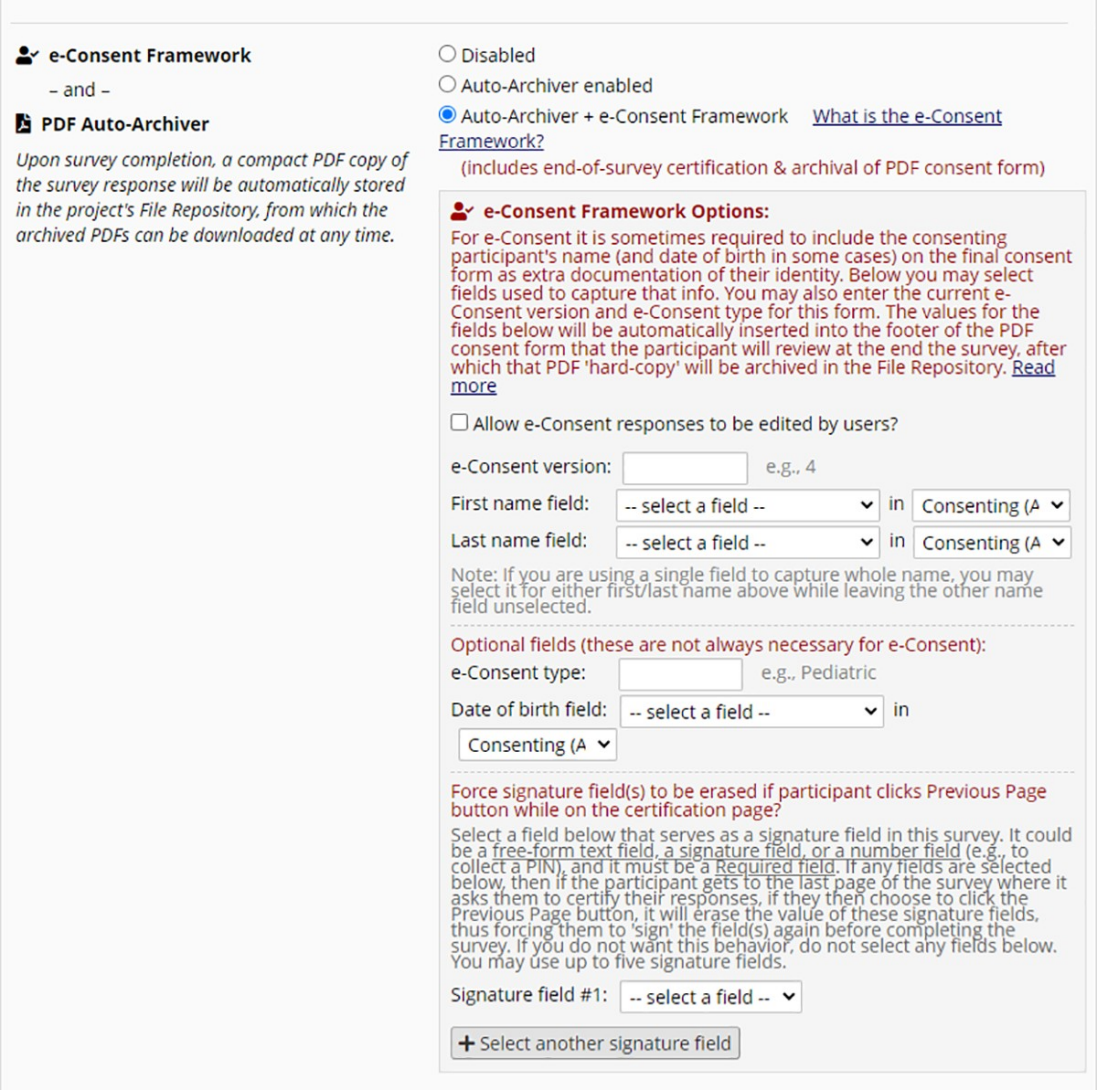
Auto archiver & eConsent framework ENABLED in survey setting, to capture all responses in a pdf, and maintain versioning of consent documents.

Historically, informed consent choices are not usually digitalised, especially where a general consent is a single agreement for study participation that is a prerequisite for a participant to join a study. Increasingly, however, informed consent processes are becoming more nuanced, and participants are provided with tiered options that require multiple responses. We have used a tiered consent approach, as described by Nembaware *et al*. in [12], for the template, recognising that not all tiers of the consent will be applicable to all studies. The design of the template is such that consent questions may be excluded or retained and the text may be altered; and the response to each tier of consent can be electronically captured and digitalised to ensure appropriate secondary use of collected data. Using the e-consent framework within REDCap allows for consent forms to be structured as surveys. ‘Auto archiver + e-consent framework’ must be selected in order to capture all consent responses in a pdf and maintain versioning of consent documents (Fig 1). The consent process could then be documented online or offline, according to that research study’s ethics approval. The consent administration could be facilitated as a split process whereby the relevant answers were documented online and then be printed to have original signatures or thumbprints in ink, if so required.

### Defining core modules and discretionary or specialised modules

Core modules were identified by exploring the overlap between multiple different bespoke TB clinical research databases (Module Index table) and identifying commonly captured elements, as described by Lew *et al*. [13]. Essential, or primary TB data fall into four main categories, which are: i) the informed consent framework which can be customized for adults or children of consenting age, ii) study data e.g. participant demographics, iii) clinical symptoms/presentation grouped as medical information e.g. medical history, TB screening, body system examinations and clinical observations; and iv) diagnostics such as imaging and laboratory test results. Supporting data that were not applicable to all studies and were likely to be used only in a subset of future TB databases were categorized as optional, including visit scheduling and calendar functionality, clinical trial randomisation, study logistics/operations and pharmacokinetic data. Further details are provided in the form of a module index, data dictionary and eCRFs, together with some examples of the e-consent, which have been made available in the GitHub repository.

### Accommodating single and repeated measurements

Usually a REDCap build for a longitudinal research study would follow the protocol standard operating procedures: data collection and data entry would occur at a specific participant visit in accordance with the study schedule of events. Different data collection styles, such as i) data collected once off e.g. demographics, ii) data collected at each visit e.g. vital signs data, iii) data of an ongoing nature e.g. medication logs and iv) a hybrid of i, ii & iii e.g. adverse events and serious adverse events, would be captured in different REDCap projects.

**Fig 2:**
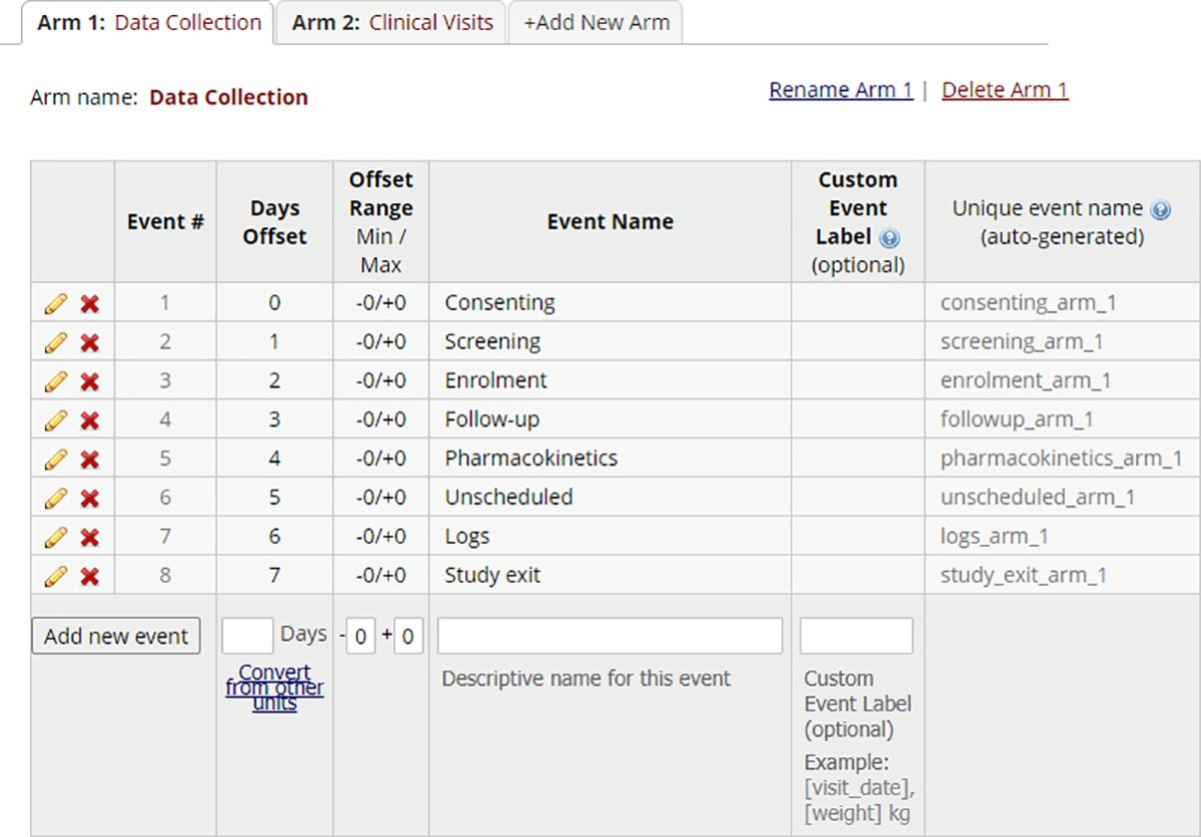
Encapsulation of data collection arm & clinical visits arm.

**Fig 3:**
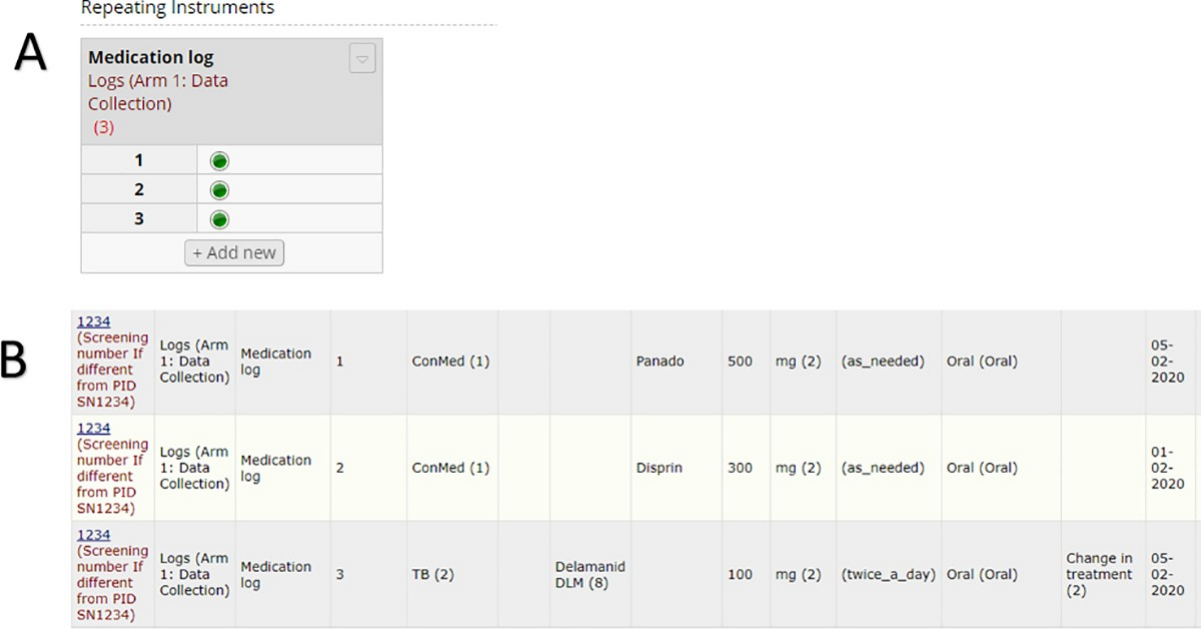
A. Example of repeating instruments for capturing medication data. B. Example of structured report for medication log, showing dummy data.

Since the aim of this project is to produce a single standardised database, however, components need to live under one umbrella but still operate independently if so needed. To accommodate the different data collection styles in a single project, we encapsulated data elements into REDCap ‘arms’ (Fig 2). Arms are assigned to participants in a randomized clinical trial where participants can be part of group A or group B. However, an arm simply implies a space where related data are stored together. Thus we created a space for data entry and clinical visits in the ‘Define My Events’ section of REDCap. Should a research project have required randomization, additional arms could be added as necessary. The Data Collection arm was further populated with specific groupings to achieve the modular functionality. By enabling the ‘Repeatable instruments and events’ feature within REDCap, instruments assigned to these groups behave in accordance to the settings and can account for all the different data collection styles (Fig 3). Data collected once is not repeated e.g. consent or screening or enrolment. Data collected at each visit is repeated as an entire event at follow-ups. Logs have repeatable single line records, and allows for multiple entries at a given event e.g. recording 4 hospital admissions or 20 different types of medications. The Clinical Visits arm was populated according to the research protocol’s standard operating procedure with no instruments allocated to this arm. Thus, scheduling of visits can occur independently without influencing the ‘Data collection Arm’.

### Data conventions

The following were implemented with the REDCap database:

#### Variable naming conventions

Descriptive names were stylized with snake_case format (e.g. date_of_birth), where possible under 26 characters as per REDCap guidance.

#### Format conventions

Date format specified as D-M-Y and Time format specified as HH: MM. Where dates are not known or the participant has difficulty with recollection, we have added an estimate checkbox. Alternatively, the date field can be broken into separate fields and the common use codes applied e.g Z89 | Unsure.

All of these items can be expanded upon by adding niche specific codes where needed, provided the base codes remain unchanged. In other words, existing codes should not be altered if they have already been used in a production environment i.e. a project that has been deployed and is in use, but new codes can be added and given their own unique code which does not conflict with existing codes.

The ‘z’ component was added to accommodate the use of these codes in numeric data points where strict validations have not been applied. Capturing ‘99’ alone for a data collection point which contains numbers could be misinterpreted as actual data e.g. age data element input as 90 but means ‘Rather not say’. A data code is a numeric or alphanumeric code that is applied to common responses, such as assigning 90 to the phrase ’Rather not say’, and is used to simplify responses to questions instead of having extensive free text answers, which would vary from person to person entering data, and not necessarily have the same meaning. However, this is an illustration of how such coding might work; actual coding is dependent on the data conventions applied in each research study. Further use of common standards and codes e.g. ICD 10 or ATC ontologies [14], facilitates easy reference amongst researchers instead of their having to memorize additional codes. Additional ontologies can be linked as lookups if enabled accordingly, although it should be noted that using large search ontologies could have an effect on system responsiveness.

## Use case scenarios

Below we have detailed two use case scenarios with feedback from those involved in using the template for their research projects.

### Use case 1

TBDBT was used to construct a database for a randomized control phase-3 TB trial with a 2x2 factorial design. This is a superiority trial testing an intensified TB treatment strategy in HIV-positive patients admitted to hospital with a new diagnosis of tuberculosis, compared to the standard of care. The study will enrol 850 patients over three years and is currently in the development phase. Data collection will revolve around TB, HIV, pharmacy data and laboratory test results. Thus, all TBDBT essential modules should be in use, specifically: informed consent, data collection, laboratory data, and logs (pharmacy data). Additional optional modules used are randomization, TB drug susceptibility testing results, adherence and contact log, were also enabled. Given the nature of the use case, HIV history is also an essential element of the database.

### Use case 1: Researcher user experience

The researcher had no experience working with REDCap prior to using the template, but reported finding TBDBT intuitive and easy to use. Some of the modules were trimmed to align with the planned research project, and all modules were used except for visual, TB Iris, Electrocardiogram, Encounters log and Lymphadenopathy log. The informed consent module was reported to be easy to use and customise. Data are being entered directly via the interface without using hard copy CRFs. Benefits reported by this user were that the hard work thinking through all the possible useful datapoints and implementing them in a database was already done, so the only actions required were to trim what was not needed. This is a new project that is starting up.

### Use case 2

A database was constructed using TBDBT for a study to evaluate the utility of an array of biomarkers in quantifying mycobacterial load in the body longitudinally on TB treatment. The initial phase is set up of study logistics for repeat visits and sample submission for testing; and selection of biomarkers for analysis. Modules used are: informed consent participant information, clinical observational data and laboratory test results, with collection of medical and TB history, participant follow up visits and per visit information. The logs functionality is used to track tests required, sample collection, sample processing and test results. Biomarker results [15] include bespoke data fields that are added to this database.

### Use case 2: Researcher user experience

The researcher had minimal REDCap experience, having once before been assisted in setting up a project, and found setting up their database using TBDBT very intuitive and quite easy to follow. The project did not require all modules, and the researcher used the template platform provided to tailor the template to the project by excluding modules that were not needed. The informed consent module is not yet in use pending acquisition of enough touch-screen devices for a COVID-19 compliant paperless consent process, but data are entered directly in an almost paperless process. The researcher has started to collect participant data and reports the excellent flow of the study processes as a highlight of TBDBT.

## Discussion

Across all research domains that rely on data collection, efforts are underway to develop data standards and ontologies that can ensure datasets are harmonised. This in turn can facilitate meta-analyses which combine data from multiple studies. The TBDBT aims to provide a template that can facilitate the standardised and harmonised collection of clinical phenotypes for TB clinical research studies, using the well-supported REDCap data platform that is already in common use for clinical research. The use of a modularised approach means that databases can be built for different types of studies and data using this single template. Furthermore, the template may be modified through addition of modules or bespoke data fields by users as they build their own databases–without disrupting the standardised collection and storage of common TB characteristics. An important component of this database is the collection of each participant’s informed consent choices, in the informed consent module: this means that records can easily be selected for secondary analyses and meta-analyses based on whether consent was given for such onward use of the data. Future development of the template will include exploring automated inputs from various external sources which would directly populate into instruments within the database, for example automated population of research data from electronic health records where appropriate consent is provided.

## Abbreviations

ATC: Anatomical Therapeutic Chemical
CDASH: Clinical Data Acquisition Standards Harmonization
CDISC: Clinical Data Interchange Standards Consortium
CRF: Case Report Form
ICD10: International Classification of Diseases and Related Health Problems (10th revision
LOINC: Logical Observation Identifiers Names and Codes
RedCap: Research Electronic Data Capture
TB: Tuberculosis
TBDBT: TB DataBase Template
